# The Association of Lipoprotein(a) and Circulating Monocyte Subsets with Severe Coronary Atherosclerosis

**DOI:** 10.3390/jcdd8060063

**Published:** 2021-06-01

**Authors:** Olga I. Afanasieva, Anastasya Yu. Filatova, Tatiana I. Arefieva, Elena A. Klesareva, Alexandra V. Tyurina, Natalia V. Radyukhina, Marat V. Ezhov, Sergei N. Pokrovsky

**Affiliations:** 1 Institute of Experimental Cardiology, National Medical Research Center of Cardiology, Ministry of Health of the Russian Federation, 121552 Moscow, Russia; AYFilatova@cardio.ru (A.Y.F.); TIArefieva@cardio.ru (T.I.A.); hea@mail.ru (E.A.K.); nradukhina@mail.ru (N.V.R.); dr.pokrovsky@mail.ru (S.N.P.); 2 A.L. Myasnikov Institute of Clinical Cardiology, National Medical Research Center of Cardiology, Ministry of Health of the Russian Federation, 121552 Moscow, Russia; alex.tyurina.cardio@yandex.ru (A.V.T.); marat_ezhov@mail.ru (M.V.E.)

**Keywords:** lipoprotein(a), hyperlipoproteinemia(a), monocyte subset, non-classical CD14+CD16++ monocyte, intermediate CD14++CD16+ monocyte, inflammation, atherosclerosis, coronary artery disease, multivessel coronary disease, autoantibodies

## Abstract

Background and aims: Chronic inflammation associated with the uncontrolled activation of innate and acquired immunity plays a fundamental role in all stages of atherogenesis. Monocytes are a heterogeneous population and each subset contributes differently to the inflammatory process. A high level of lipoprotein(a) (Lp(a)) is a proven cardiovascular risk factor. The aim of the study was to investigate the association between the increased concentration of Lp(a) and monocyte subpopulations in patients with a different severity of coronary atherosclerosis. Methods: 150 patients (124 males) with a median age of 60 years undergoing a coronary angiography were enrolled. Lipids, Lp(a), autoantibodies, blood cell counts and monocyte subpopulations (classical, intermediate, non-classical) were analyzed. Results: The patients were divided into two groups depending on the Lp(a) concentration: normal Lp(a) < 30 mg/dL (*n* = 82) and hyperLp(a) ≥ 30 mg/dL (*n* = 68). Patients of both groups were comparable by risk factors, autoantibody levels and blood cell counts. In patients with hyperlipoproteinemia(a) the content (absolute and relative) of non-classical monocytes was higher (71.0 (56.6; 105.7) vs. 62.2 (45.7; 82.4) 10^3^/mL and 17.7 (13.0; 23.3) vs. 15.1 (11.4; 19.4) %, respectively, *p* < 0.05). The association of the relative content of non-classical monocytes with the Lp(a) concentration retained a statistical significance when adjusted for gender and age (r = 0.18, *p* = 0.03). The severity of coronary atherosclerosis was associated with the Lp(a) concentration as well as the relative and absolute (*p* < 0.05) content of classical monocytes. The high content of non-classical monocytes (OR = 3.5, 95% CI 1.2–10.8) as well as intermediate monocytes (OR = 8.7, 2.5–30.6) in patients with hyperlipoproteinemia(a) were associated with triple-vessel coronary disease compared with patients with a normal Lp(a) level and a low content of monocytes. Conclusion: Hyperlipoproteinemia(a) and a decreased quantity of classical monocytes were associated with the severity of coronary atherosclerosis. The expansion of CD16+ monocytes (intermediate and non-classical) in the presence of hyperlipoproteinemia(a) significantly increased the risk of triple-vessel coronary disease.

## 1. Introduction

Lipoprotein(a), discovered in 1963 by Norwegian scientist Kare Berg, belongs to the family of apoB100-containing lipoproteins and represents a supramolecular complex encompassing a low-density lipoprotein (LDL)-like particle and a highly glycosylated, highly polymorphic protein, apolipoprotein(a).

Numerous pathophysiological, epidemiological and genetic studies of the role of lipoprotein(a) as well as randomized controlled clinical trials over the past decades have proven the causal association of an increased Lp(a) concentration with any atherosclerotic cardiovascular diseases, calcifying aortic valve stenosis and complications after myocardial revascularization [1,2].

Today, we can consider elevated levels of Lp(a)—hyperlipoproteinemia(a) (hyperLp(a)) as the most common genetic lipid disorder, occurring in about 20% of the population, which is about 1.4 billion people worldwide [1]. HyperLp(a) is associated with manifestations of atherothrombotic complications starting from childhood and adolescence [3], heart attacks at a young age [4] and the development of aortic valve stenosis at an older age [5]. An elevated Lp(a) concentration is one of the most obvious residual risk factors for cardiovascular complications in patients who have achieved the target levels of low-density lipoprotein cholesterol (LDL-C) on lipid-lowering therapy [6,7,8].

Currently, there are no specific treatments for Lp(a) level reduction and the most effective and promising ones are still at the stage of clinical trials [9]. Specific Lp(a) apheresis and LDL apheresis have demonstrated the stabilization, and even the regression, of atherosclerotic plaques in the coronary and carotid arteries as well as a significant reduction in the risk of adverse cardiovascular events [10,11].

According to modern concepts, chronic inflammation associated with the uncontrolled activation of innate and acquired immunity plays a fundamental role at all stages of atherogenesis. The local inflammatory reactions are present from the earliest stages of the atherosclerotic lesion formation and contribute to plaque destabilization [12]. Several types of immune cells, primarily monocytes as well as T- and B-lymphocytes are implicated in the inflammatory process in atherosclerosis. Monocytes are a heterogeneous population and each subset contributes differently to the inflammatory process. According to the phenotype (CD14 and CD16 exposure on the cell membrane) and functional properties, monocytes are identified as classical (85–90% of the total population of monocytes in healthy subjects), intermediate (5% of the total population of monocytes) and non-classical (10% of the total population) subsets [13]. A distinctive feature of classical monocytes is the expression of chemokine CCR2 receptors and a high phagocytic activity [14]. Non-classical monocytes are characterized by the expression of CX3CR1 [14,15]; they “patrol” the vessel wall and differentiate mainly into “anti-inflammatory” macrophages during migration into the tissues. The intermediate subset is considered to be the most atherogenic due to an increased ability to synthesize inflammatory cytokines [14]. Classical human monocytes can consistently differentiate into an intermediate subset and then into a non-classical subset [16].

To date, there are no unequivocal answers to the question of how the circulating monocyte subsets affect the processes occurring in the vessel wall [17]. According to several studies, the content of CD16+ monocytes is associated with an increased cardiovascular risk [18].

Earlier, we demonstrated the relationship between changes in the immunological balance, an elevated Lp(a) level and the development of severe and rapidly progressive atherosclerosis in men [19]. This study aimed to investigate the association between the increased plasma Lp(a) concentration and monocyte subpopulations in patients with a different severity of coronary atherosclerosis.

## 2. Materials and Methods

The study included 150 adult patients with a clinical indication for coronary angiography who gave informed consent to participate. All patients enrolled in this study had been receiving statins and acetylsalicylic acid for at least one month before the enrollment and beta-blockers and/or angiotensin-converting enzyme (ACE) inhibitors/angiotensin receptor blockers if indicated. Stenotic atherosclerosis was identified as the narrowing of the artery lumen by more than 50%. The exclusion criteria were an acute coronary syndrome, infectious and inflammatory diseases in the previous three months, congestive heart failure of the III–IV functional class, systemic diseases of the connective tissue, chronic kidney disease of stage IV or V, severe thyroid dysfunction (thyroid stimulating hormone two times lower than the lower limit or two times higher than the upper limit), acute hepatitis, liver cirrhosis and Lp(a)-lowering therapy (apheresis, nicotinic acid, PCSK9 inhibitors, glucocorticoids, sex hormones).

The lipids, Lp(a), autoantibodies (autoAbs) against apoB100-containing lipoproteins and CRP were analyzed in serum of all of the patients. The concentration of total cholesterol (TC), triglycerides (TG) and high-density lipoprotein cholesterol (HDL-C) was measured by the enzymatic colorimetric method on an Architect C-8000 analyzer (Abbott, Chicago, IL, USA). The level of low-density lipoprotein cholesterol (LDL-C) was calculated using Friedewald’s formula with modifications [20]:

LDL-Ccorr (mM/L) = TC − HDL-C − TG/2.2 − 0.3 × Lp(a) mass (md/dL)/38.7

where LDL-Ccorr is the level of LDL-C corrected to the level of Lp(a)-cholesterol. The Lp(a) concentration was measured by enzyme immunoassay (ELISA) using monospecific polyclonal sheep antibodies against human Lp(a) [21]. The method was validated with two kits, TintElize Lp(a) (Biopool AB, Umea, Sweden) and Immunozym Lp(a) (Progen Biotechnik GmbH, Heidelberg, Germany). The control serum (Technoclone, Vienna, Austria) was approved by the International Federation of Clinical Chemistry and was used to standardize the ELISA. AutoAbs against Lp(a) and LDL were detected by ELISA according to a previously developed method [22]. The level of C-reactive protein (hsCRP) was determined using a highly sensitive ELISA kit (Vector-Best, Novosibirsk, Russia).

The lymphocyte-monocyte index was calculated as the ratio of the absolute numbers of blood lymphocytes and monocytes. The immunophenotyping of monocytes was performed in the blood samples by direct immunofluorescence using fluorescently labeled antibodies to CD14, CD16 antigens (Beckman Coulter, Brea, CA, USA) and a lysing solution (BD Immunocytometry Systems) following the manufacturer’s manuals. The samples were analyzed on FACS Calibur and FACS Canto flow cytometers (BD Immunocytometry Systems). The monocytes were gated according to forward sight scatter parameters and the monocyte subsets were identified as classical (CD14++CD16−), intermediate (CD14++CD16+) and non-classical (CD14+CD16++) according to the routinely used protocol (Figure S1 in the Supplementary Materials) [23].

The descriptive statistics of the continuous quantitative variables are presented as the median (25th and 75th percentile) or a 95% confidential interval. The analytical statistics were performed using Mann–Whitney and Kruskal–Wallis tests. To compare the frequency indicators between the groups, the χ^2^ criterion was used. To assess the significance of the association between the parameters studied and the presence and severity of stenotic atherosclerosis, the odds ratio (OR) with a 95% confidence interval (CI) was calculated. To analyze the relationship of the studied parameters, the non-parametric Spearman correlation analysis method was used. The threshold values of the monocyte content associated with the presence of hyperLp(a) were obtained by the analysis of the curves of operational characteristics (ROC analysis). A multivariate regression analysis and a logistic regression analysis were performed to identify the association between the concentration of Lp(a) and the monocyte subset distribution as well as their relationship with stenotic atherosclerosis. The risk factors that demonstrated an association with stenotic atherosclerosis in a single-factor correlation analysis as well as those traditionally associated with atherosclerosis were introduced into the model. When creating the model, the absence of internal correlations between the estimated parameters was also taken into account. The differences were considered statistically significant at *p* < 0.05.

## 3. Results

The concentration of Lp(a) in the cohort of examined patients varied in a very wide range; the distribution histogram was characteristically shifted to the left (Figure S2 in the Supplementary Materials). The patients were divided into two groups based on Lp(a) concentrations greater than or less than 30 mg/dL. The groups did not differ by age, sex, history of hypertension, diabetes, smoking or other risk factors (Table 1).

There were no significant differences in the content of the total blood leukocytes and leukocyte subtypes, the concentration of hsCRP, circulating immune complexes and the content of autoAbs against atherogenic apoB100-containing lipoproteins (Table 2).

**Figure 1:**
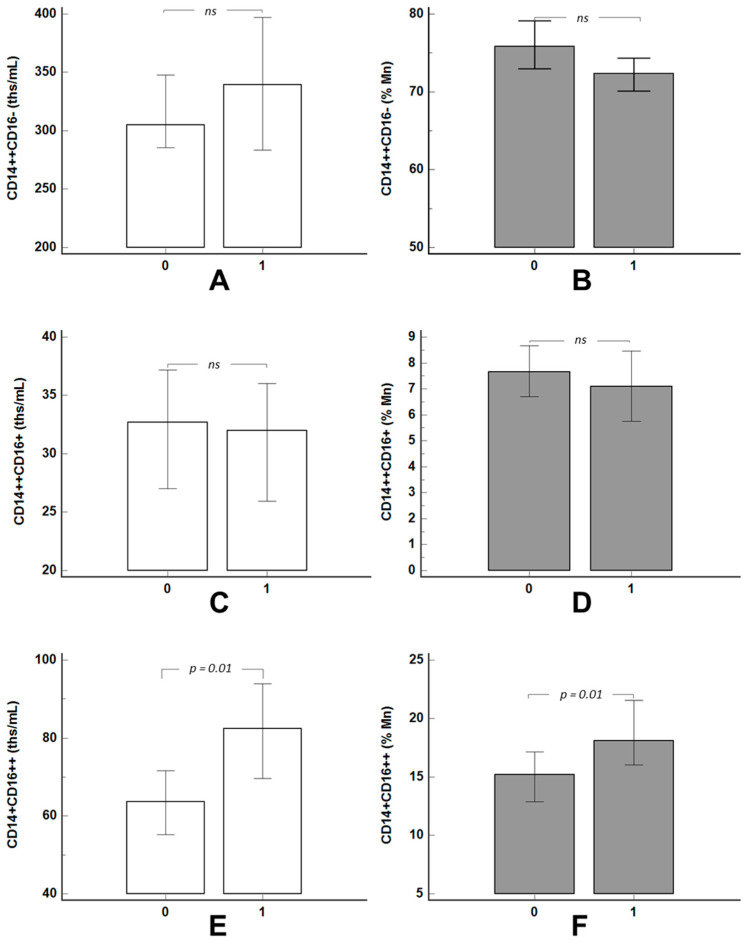
Absolute (**A**,**C**,**E**) and relative (**B**,**D**,**F**) content of the monocyte subsets in the groups of patients with (“1”) and without (“0”) hyperlipoproteinemia(a). Data are presented as a median and a 95% confidential interval for the median. Mn = monocyte content. “ns”—not significant (*p* > 0.05).

In patients with hyperLp(a) the number of non-classical CD14+CD16++ monocytes (relative and absolute counts) were higher (Figure 1A,B). There were no significant differences in the content of other monocyte subsets (Figure 1C–F).

A weak positive correlation was found between the Lp(a) concentration and the content of non-classical CD14+CD16++ monocytes in absolute and relative values (r = 0.20, *p* = 0.01 and r = 0.19, *p* = 0.02, respectively) in the total cohort of patients.

According to the ROC analysis, the content of non-classical CD14+CD16++ monocytes of more than 67.6 10^3^/mL and 21.3% of monocytes was associated with hyperLp(a) with a sensitivity of 67% and 40%, respectively, and a specificity of 58% and 82%, respectively. No significant associations were found for the other monocyte subsets.

A slight negative correlation was found between age and the absolute number of classical CD14++CD16− monocytes (r = −0.301, *p* = 0.0002). The association of the relative content of classical (r = −0.26, *p* = 0.0013), intermediate (r = 0.247, *p* = 0.002) and non-classical monocytes (r = 0.169, *p* = 0.04) with age was also observed.

According to the multivariate regression analysis, the association of the relative content of non-classical CD14+CD16++ monocytes with the Lp(a) concentration retained a statistical significance when adjusted for gender and age (r = 0.176, *p* = 0.03).

**Figure 2:**
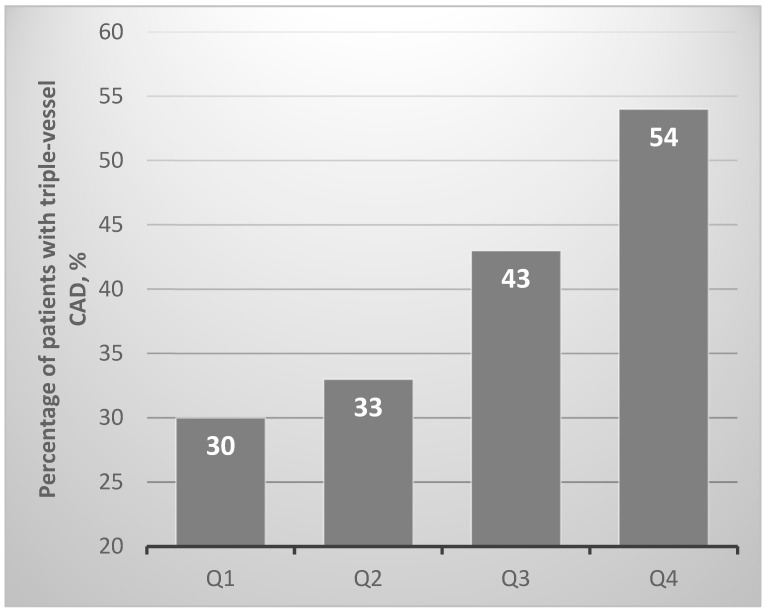
Percentage of patients with triple-vessel coronary disease (CAD) in accordance with Lp(a) quartiles, mg/dL: Q1 < 7.2, Q2 from 7.2 to 24.3; Q3 from 24.4 to 67.8; Q4 ≥ 67.8.

The concentration of Lp(a) of the upper quartile of the distribution was associated with the presence of triple-vessel coronary disease (CAD) with an OR of 4.24 (1.26–14.18), *p* = 0.02. In this subgroup, a triple-vessel lesion occurred in more than 50% of patients (Figure 2).

**Figure 3:**
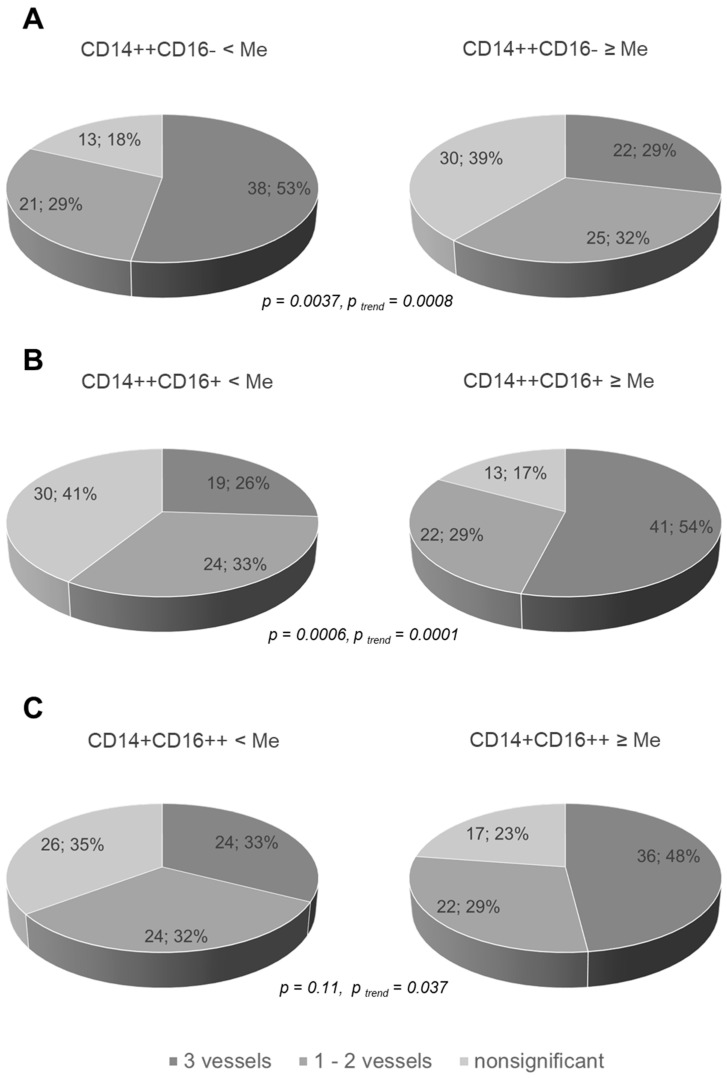
Coronary atherosclerosis and the percentage of the monocyte subsets. Data are presented as absolute numbers and the percentage of patients with a varying severity of coronary artery disease depending on the relative of the monocyte subset below and above the median. The median value for classic CD14++CD16− monocytes was 73.6%, intermediate CD14++CD16+ was 7.3% and non-classical CD14+CD16++ was 16.4%. (**A**)—classical CD14++CD16−, (**B**)—intermediate CD14++CD16+ and (**C**)—non-classical CD14+CD16++ monocytes. Me = median.

According to the median distribution of the monocyte subsets, triple-vessel CAD was more common in patients with a lower percentage of classical CD14++CD16− monocytes (values below the median, Figure 3A) and with a higher percentage of non-classical CD14+CD16++ and intermediate CD14++CD16+ monocytes (Figure 3B,C). No significant differences in the absolute values were found.

In a multiple regression analysis adjusted for gender and age, an increase in the severity of the coronary lesions was independently associated with the concentration of Lp(a) as well as the relative (r = −0.16, *p* < 0.05) and absolute (r = −0.21, *p* < 0.05) content of classical CD14++CD16− monocytes. According to the logistic regression analysis adjusted for gender and age, hyperLp(a) and the content of intermediate CD14++CD16+ and classical CD14++CD16− monocytes were differentially associated with triple-vessel CAD (Table 3). After an adjustment for hyperLp(a), these immune parameters remained independently associated with multivessel lesions of the coronary arteries. After an adjustment for the presence of diabetes mellitus, arterial hypertension and smoking, all parameters excepting hyperLp(a) and the percentage of intermediate CD14++CD16+ monocytes lost their significance as a risk factor for triple-vessel CAD.

**Figure 4:**
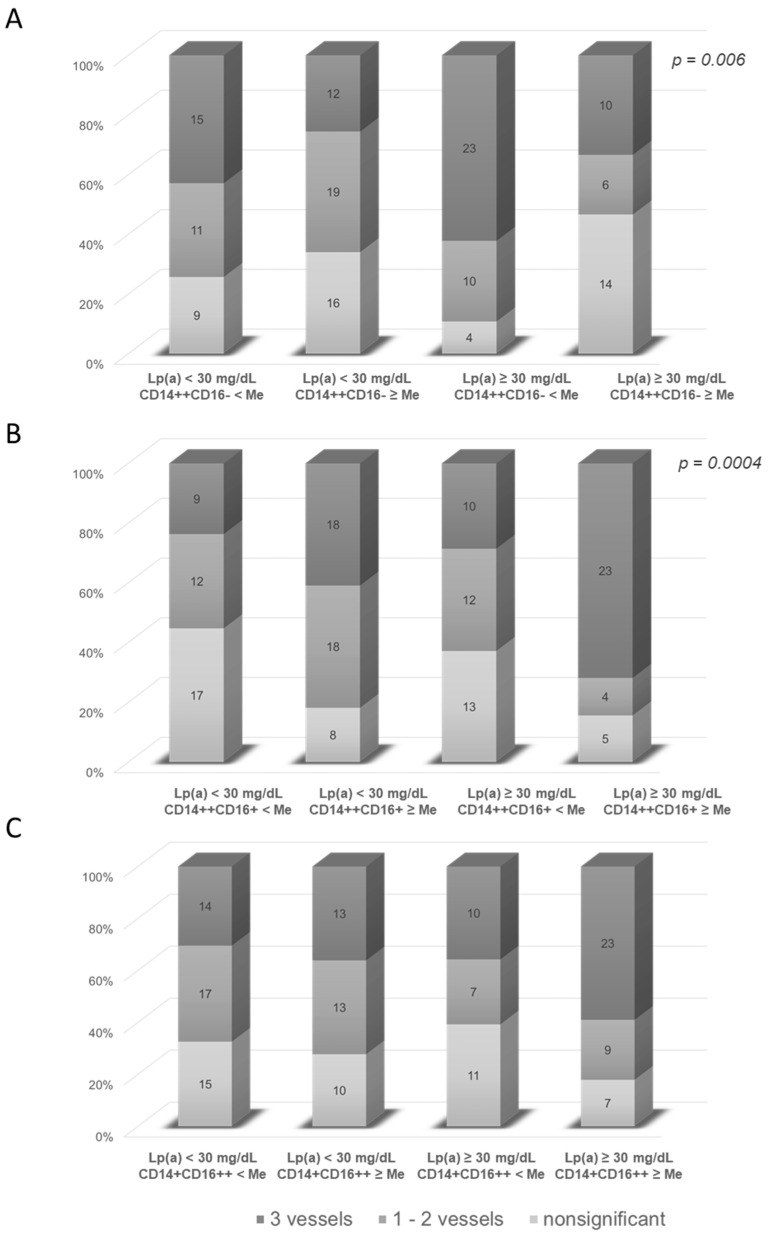
Severity of coronary artery disease depending on the presence of hyperLp(a) and the relative content of classical CD14++CD16− (**A**), intermediate CD14++CD16+ (**B**) and non-classical CD14+CD16++ monocytes (**C**). The data are presented as the percentage of patients and the absolute number of patients (numbers within the bars) with a different severity of coronary atherosclerosis in the subgroups depending on the combination of normal (<30 mg/dL) and increased (≥30 mg/dL) concentrations of Lp(a) as well as the relative content of monocytes below and above the median. The corresponding values of the median for the relative (% of the total number of monocytes) content of classical CD14++CD16−, intermediate CD14++CD16+ and non-classical CD14+CD16++ subpopulations of monocytes correspond to the values indicated in Table 3. Me = median.

To assess the mutual influence of Lp(a) and the monocyte subsets on the severity of coronary atherosclerosis, patients were divided according to the combination of absolute/percentage values of the monocyte subsets and the Lp(a) concentration. We found the contribution of both hyperLp(a) and a decreased percentage of classical monocytes/increased content of intermediate monocytes in the presence of triple-vessel CAD (Figure 4). For the absolute content of the monocyte subset, the same trend was found (Figure S3, Supplementary Materials).

HyperLp(a) and an increased content of CD16+ monocytes were strongly associated with the presence of triple-vessel CAD (Table 4).

It should be noted that the high content of non-classical CD14+CD16++ monocytes as well as intermediate CD14++CD16+ monocytes and the increased concentration of Lp(a) were associated with triple-vessel CAD compared with patients with single- or double-vessel disease (OR = 3.1 (95% CI 1.1–8.8) and OR = 7.7 (95% CI 2.0–30.1), respectively).

## 4. Discussion

The physiological role of Lp(a) as well as the mechanisms that contribute to its high atherogenicity are ambiguous. It is assumed that the formation of Lp(a)-containing circulating immune complexes [24] and their interaction with macrophages [25] may be possible pathogenetic mechanisms leading to the development of inflammation in the vessel wall. The association of autoAbs to Lp(a) as well as the lymphocyte activation marker sCD25 with the presence and severity of coronary atherosclerosis that we previously showed [19] may indicate the contribution of Lp(a) to atherogenesis by the activation of humoral and cellular immunity.

The discovery of the key role of monocytes/macrophages in the development of atherosclerosis [26] was a background for the study of blood monocyte subsets in patients with a cardiovascular pathology. According to a few reports, the high levels of CD16+ monocytes were associated with hypercholesterolemia and unstable coronary atherosclerotic plaques [27]. Intermediate CD14++CD16+ monocytes were shown to predict cardiovascular events in patients with chronic kidney disease [28] and a broad patient population at cardiovascular risk [18]. At the same time, an increased content of intermediate CD14++CD16+ monocytes was associated with a better prognosis in patients with heart failure [29]. The increased content of non-classical CD14+CD16++ monocytes and the reduced content of classical CD14++CD16− monocytes were detected in patients with high levels of small dense LDL [30] and patients with “dysfunctional” small high-density lipoproteins [31]. The same researchers found an increased content of intermediate CD14++CD16+ monocytes in patients with stable CHD with an Lp(a) plasma level > 50 mg/dL [32].

In this study, we showed for the first time the association between the increased concentration of Lp(a) (≥30 mg/dL) and the high content of non-classical CD14+CD16++ monocytes in absolute and relative counts. The increased percentage of non-classical CD16+ monocytes in patients with hyperLp(a) was associated with the elevated risk of multivessel CAD.

The contribution of non-classical monocytes to chronic inflammation has been actively discussed in recent years. It was demonstrated that non-classical CD14+CD16++ monocytes can differentiate into inflammatory macrophages and play a key role in joint inflammation in a mouse model of arthritis [33]. Non-classical monocytes can also differentiate into osteoclasts [34]. In mice models of autoimmune diseases, non-classical monocytes stimulated B-cells, indicating their contribution to the production of autoantibodies [35]. This effect may be mediated by the activation of IL-4 synthesis by T-cells as was revealed by [36].

According to other authors, the main function of non-classical monocytes is “patrolling” the endothelium, recognizing viruses, “cell debris” or other damage signals [37]. These cells are ready to transmigrate through the endothelial layer and genes associated with cytoskeletal motility are mainly expressed in non-classical monocytes [38].

Urbanski et al. showed that the content of non-classical monocytes in the blood is associated with endothelial dysfunction regardless of other risk factors of atherosclerosis. Patients with CAD and the high frequency of non-classical CD14+CD16++ monocytes (the upper tertile of distribution) presented an impaired vascular response to acetylcholine administration and a significantly increased superoxide production compared with patients with a low non-classical monocyte content [39].

Several studies have demonstrated the relationship between an increased Lp(a) concentration and multivessel atherosclerosis [40]. Lp(a) was proven to promote endothelial dysfunction and damage via diverse mechanisms [41]. It can be assumed that the expansion of non-classical CD14+CD16++ monocytes in patients with hyperLp(a) is related to their “innate” control over tissues to detect damaged cells including an Lp(a)-damaged endothelium.

There is evidence that intermediate monocytes have the most pronounced pro-inflammatory properties. These cells can produce high cytokine levels upon stimulation [38]. The subset of intermediate monocytes expresses high levels of surface markers involved in interactions with T-cells and thus can effectively stimulate T-cell proliferation [42,43]. The association we established between the increased relative content of the intermediate monocytes and triple-vessel CAD regardless of Lp(a) concentration indicated that the redistribution of monocytes from classical to “pro-inflammatory” CD16+ subsets may be mediated by other atherogenic lipoproteins; in particular, the most atherogenic subfractions of small dense LDL. The combination of an atherogenic LDL profile, i.e., the presence of small dense lipoproteins, with an increased concentration of Lp(a) was associated with a significantly increased risk of CHD [44]. On the other hand, the CD14++CD16+ monocyte content was associated with the vulnerability of coronary plaques in CHD patients with corrected LDL levels [45]. These data suggest the existence of mechanisms independent of the lipid profile that can lead to the expansion of pro-inflammatory monocytes and contribute to atherosclerosis progression.

Understanding the fundamental regulation of the differentiation and functioning of monocytes in the presence of various types of lipid disorders may help in determining the directions of future therapeutic interventions aimed at blocking or, on the contrary, activating the components of the immune system.

## 5. Limitations of the Study

We included patients admitted to a single center with a diagnosis of coronary artery disease for an additional examination and the determination of further treatment strategy so there was a high proportion of subjects with severe stenotic atherosclerosis and a few of them were under 55 years of age.

The relationship between the extent of peripheral atherosclerosis and the studied parameters was not analyzed.

For monocyte subset immunophenotyping, anti-CD14 and anti-CD16 antibodies were used. Although this approach is widely accepted, there is an opinion that this is not enough.

## 6. Conclusions

We found for the first time an association between Lp(a) concentration and the blood content of non-classical CD14+CD16++ monocytes regardless of gender and age. The increased level of Lp(a) and the decreased quantity of classical CD14++CD16− monocytes were associated with the severity of coronary atherosclerosis. Both hyperLp(a) and a higher content of intermediate CD14++CD16+ monocytes were predisposing factors for triple-vessel coronary disease regardless of gender, age or other risk factors. The expansion of CD16+ monocytes (intermediate and non-classical) in the presence of hyperLp(a) significantly increased the risk of triple-vessel coronary disease. Further studies on the differentiation and functioning of the monocyte subsets in the presence of different lipid disorders, especially hyperLp(a), are needed.

## Figures and Tables

**Table 1:** Characteristics of the study patients.

	Lp(a) < 30 mg/dL *n* = 82	Lp(a) ≥ 30 mg/dL *n* = 68	*p*
Males	67 (81%)	47 (69%)	0.17
Age, years	61 (56; 64)	58 (52; 65)	0.23
Body mass index, kg/m^2^	29 (26; 32)	28 (26; 33)	0.94
Type 2 diabetes	14 (17%)	10 (15%)	0.76
Glucose, mM/L	5.6 (5.3; 6.0)	5.7 (5.3; 6.5)	0.25
Smoking	28 (34%)	20 (29%)	0.62
Family history of CHD	7 (9%)	6 (9%)	0.89
Stenotic atherosclerosis of coronary arteries	51 (62%)	40 (59%)	0.96
Lp(a), mg/dL	8.2 (3.6; 13.4)	73.6 (42.0; 107.1)	<0.0001
TC, mM/L	4.3 (3.4; 5.6)	4.5 (3.8; 5.5)	0.52
TG, mM/L	1.5 (1.2; 2.1)	1.2 (0.9; 1.8)	0.03
HDL-C, mM/L	1.0 (0.9; 1.3)	1.1 (0.9; 1.3)	0.30
LDL-C, mM/L	2.5 (1.7; 3.9)	2.7 (2.2; 4.0)	0.25
LDL-Ccorr, mM/L	2.3 (1.6; 3.8)	2.2 (1.6; 3.5)	0.36

The data are presented as a median (25%; 75%) or n (%).

**Table 2:** Immune parameters in association with the lipoprotein(a) level.

	Lp(a) < 30 mg/dL		Lp(a) ≥ 30 mg/dL	
	Median	IQR	Median	IQR
Leukocytes, 10^6^/mL	7.2	5.9–8.4	7.4	5.0–8.6
Lymphocytes, 10^6^/mL	2.0	1.4–2.5	2.1	1.67–2.58
Monocytes, 10^6^/mL	0.42	0.34–0.56	0.47	0.35–0.62
Monocyte-lymphocyte index	0.23	0.18–0.31	0.23	0.17–0.27
hsCRP, mg/L	1.2	0.80–2.20	3.6	1.48–4.10
Circulating immune complex, lab. unit	82.8	64.7–119.3	85.0	66.7–107.7
IgG autoAbs against, lab. unit.				
LDL	20.5	12.0–38.0	26.0	12.3–46.4
oxLDL	28.0	15.5–40.7	29.7	17.2–50.6
Lp(a)	27.4	21.1–33.4	27.5	21.3–30.5
oxLp(a)	46.2	36.3–55.2	42.6	28.7–50.5
IgM autoAbs against lab. unit.				
LDL	12.2	8.6–19.5	16.3	9.6–43.1
oxLDL	22.2	18.2–26.4	26.6	19.6–51.6
Lp(a)	17.4	11.7–22.7	17.5	12.6–30.3
oxLp(a)	27.6	13.8–43.2	29.9	15.9–43.0

The data are presented as a median (25%; 75%). Me = median; IQR = interquartile range 25%; 75%; autoAbs = autoantibodies.

**Table 3:** HyperLp(a) and the monocyte subsets in association with triple-vessel coronary disease.

Parameter	OR	95% CI
**Adjusted for gender and age**		
Lp(a) ≥ 30 mg/dL	2.28 *	1.13–4.61
Classical CD14++CD16− Mn ≥ 73.6 (% from monocytes)	0.41 *	0.20–0.82
Classical CD14++CD16− Mn ≥ 327.4 (10^3^/mL)	0.95	0.46–1.99
Intermediate CD14++CD16+ Mn ≥ 7.3 (% from monocytes)	2.99 *	1.46–6.13
Intermediate CD14++CD16+ Mn ≥ 32.5 (10^3^/mL)	1.38	0.69–2.76
Non-classical CD14+CD16++ Mn ≥ 16.4 (% from monocytes)	1.75	0.88–3.48
Non-classical CD14+CD16++ Mn ≥ 70.2 (10^3^/mL)	1.20	0.60–2.40
**Adjusted for gender, age, Lp(a) ^**		
Lp(a) ≥ 30 mg/dL	2.05 *	1.00–4.20
Classical CD14++CD16− Mn ≥ 73.6 (% from monocytes)	0.45 *	0.22–0.92
Lp(a) ≥ 30 mg/dL	2.35 *	1.15–4.81
Classical CD14++CD16− Mn ≥ 327.4 (10^3^/mL)	0.86	0.41–1.79
Lp(a) ≥ 30 mg/dL	2.34 *	1.15–4.77
Intermediate CD14++CD16+ Mn ≥ 32.5 (10^3^/mL)	1.42	0.69–2.89
Lp(a) ≥ 30 mg/dL	2.51 *	1.20–5.22
Intermediate CD14++CD16+ Mn ≥ 7.3 (% from monocytes)	3.23 *	1.54–6.79
Lp(a) ≥ 30 mg/dL	2.31 *	1.12–4.75
Non-classical CD14+CD16++ Mn ≥ 16.4 (% from monocytes)	1.03	0.50–2.11
Lp(a) ≥ 30 mg/dL	2.14 *	1.05–4.36
Non-classical CD14+CD16++ Mn ≥ 70.2 (10^3^/mL)	1.58	0.78–3.18
**Adjusted for gender, age, Lp(a) ^, smoking, diabetes mellitus, arterial hypertension**		
Lp(a) ≥ 30 mg/dL	2.49 *	1.17–5.31
Classical CD14++CD16− Mn ≥ 327.4 (10^3^/mL)	0.98	0.45–2.15
Lp(a) ≥ 30 mg/dL	2.26 *	1.06–4.83
Classical CD14++CD16− Mn ≥ 73.6 (% from monocytes)	0.54	0.25–1.14
Lp(a) ≥ 30 mg/dL	2.64 *	1.22–5.69
Intermediate CD14++CD16+ Mn ≥ 7.3 (% from monocytes)	2.88 *	1.33–6.24
Lp(a) ≥ 30 mg/dL	2.47 *	1.17–5.22
Intermediate CD14++CD16+ Mn ≥ 32.5 (10^3^/mL)	1.30	0.62–2.75
Lp(a) ≥ 30 mg/dL	2.39 *	1.12–5.07
Non-classical CD14+CD16++ Mn ≥ 16,4 (% from monocytes)	1.41	0.68–2.95
Lp(a) ≥ 30 mg/dL	2.43 *	1.14–5.20
Non-classical CD14+CD16++ Mn ≥ 70.2 (10^3^/mL)	1.12	0.53–2.38

* *p* < 0.05, ^ for Lp(a) OR are also present in the table. Mn = monocyte content.

**Table 4:** Association of triple-vessel coronary disease depending on Lp(a) level and the monocyte subsets.

	Lp(a) < 30 mg/dL Mn < Me	Lp(a) < 30 mg/dL Mn ≥ Me	Lp(a) ≥ 30 mg/dL Mn < Me	Lp(a) ≥ 30 mg/dL Mn ≥ Me
CD14++CD16− (% from Mn)	1	0.45 (0.15–1.37)	3.45 (0.90–13.24)	0.42 (0.13–1.36)
CD14++CD16+ (% from Mn)	1	4.25 (1.33–13.56) *	1.45 (0.45–4.60)	8.69 (2.46–30.63) *
CD14+CD16++ (% from Mn)	1	1.39 (0.46–4.18)	0.97 (0.32–3.00)	3.50 (1.15–10.75) *

* *p* < 0.05. Data are presented as an OR (95% CI) relative to patients with unconfirmed CHD. Mn = monocyte content; Me = median.

## Data Availability

The data presented in this study are available on request from the corresponding author.

## Supplementary Materials

The following are available online at https://www.mdpi.com/article/10.3390/jcdd8060063/s1, Figure S1: Monocyte subsets definition based on a typical distribution of events in a CD14 and CD16 staining, Figure S2: Histogram of the distribution of the Lp(a) concentration in the examined patients, Figure S3: Severity of coronary artery disease depending on the presence of hyperLp(a) and absolute content of monocyte subsets.

## References

[1] Tsimikas S., Fazio S., Ferdinand K.C., Ginsberg H.N., Koschinsky M.L., Marcovina S.M., Moriarty P.M., Rader D.J., Remaley A.T., Reyes-Soffer G. (2018). NHLBI Working Group Recommendations to Reduce Lipoprotein(a)-Mediated Risk of Cardiovascular Disease and Aortic Stenosis. J. Am. Coll. Cardiol..

[2] Afanasieva O.I., Pokrovsky S.N. (2019). Hyperlipoproteidemia(a) as a dangerous genetically determined disorder of lipid metabolism and risk factors for atherothrombosis and cardiovascular disease. Russ. J. Cardiol..

[3] Sultan S.M., Schupf N., Dowling M.M., Deveber G.A., Kirton A., Elkind M.S. (2014). Review of lipid and lipoprotein(a) abnormalities in childhood arterial ischemic stroke. Int. J. Stroke.

[4] Kamstrup P.R., Benn M., Tybjaerg-Hansen A., Nordestgaard B.G. (2008). Extreme lipoprotein(a) levels and risk of myocardial infarction in the general population: The Copenhagen City Heart Study. Circulation.

[5] Capoulade R., Yeang C., Chan K.L., Pibarot P., Tsimikas S. (2018). Association of Mild to Moderate Aortic Valve Stenosis Progression With Higher Lipoprotein(a) and Oxidized Phospholipid Levels: Secondary Analysis of a Randomized Clinical Trial. JAMA Cardiol..

[6] Willeit P., Ridker P.M., Nestel P.J., Simes J., Tonkin A.M., Pedersen T.R., Schwartz G.G., Olsson A.G., Colhoun H.M., Kronenberg F. (2018). Baseline and on-statin treatment lipoprotein(a) levels for prediction of cardiovascular events: Individual patient-data meta-analysis of statin outcome trials. Lancet.

[7] O’Donoghue M.L., Fazio S., Giugliano R.P., Stroes E.S.G., Kanevsky E., Gouni-Berthold I., Im K., Lira Pineda A., Wasserman S.M., Češka R. (2019). Lipoprotein(a), PCSK9 Inhibition, and Cardiovascular Risk. Circulation.

[8] Bittner V.A., Szarek M., Aylward P.E., Bhatt D.L., Diaz R., Edelberg J.M., Fras Z., Goodman S.G., Halvorsen S., Hanotin C. (2020). Effect of Alirocumab on Lipoprotein(a) and Cardiovascular Risk After Acute Coronary Syndrome. J. Am. Coll. Cardiol..

[9] Kosmas C.E., Sourlas A., Mallarkey G., Silverio D., Ynoa D.Y., Montan P.D., Guzman E., Garcia M.J. (2019). Therapeutic management of hyperlipoproteinemia(a). Drugs Context.

[10] Pokrovsky S.N., Afanasieva O.I., Ezhov M.V. (2016). Lipoprotein(a) apheresis. Curr. Opin. Lipidol..

[11] Schettler V.J.J., Neumann C.L., Peter C., Zimmermann T., Julius U., Hohenstein B., Roeseler E., Heigl F., Grützmacher P., Blume H. (2019). Lipoprotein apheresis is an optimal therapeutic option to reduce increased Lp(a) levels. Clin. Res. Cardiol. Suppl..

[12] Sager H.B., Nahrendorf M. (2016). Inflammation: A trigger for acute coronary syndrome. Q. J. Nucl. Med. Mol. Imaging.

[13] Ziegler-Heitbrock L., Ancuta P., Crowe S., Dalod M., Grau V., Hart D.N., Leenen P.J., Liu Y.J., MacPherson G., Randolph G.J. (2010). Nomenclature of monocytes and dendritic cells in blood. Blood.

[14] Kapellos T.S., Bonaguro L., Gemünd I., Reusch N., Saglam A., Hinkley E.R., Schultze J.L. (2019). Human Monocyte Subsets and Phenotypes in Major Chronic Inflammatory Diseases. Front. Immunol..

[15] Boyette L.B., Macedo C., Hadi K., Elinoff B.D., Walters J.T., Ramaswami B., Chalasani G., Taboas J.M., Lakkis F.G., Metes D.M. (2017). Phenotype, function, and differentiation potential of human monocyte subsets. PLoS ONE.

[16] Cignarella A., Tedesco S., Cappellari R., Fadini G.P. (2018). The continuum of monocyte phenotypes: Experimental evidence and prognostic utility in assessing cardiovascular risk. J. Leukoc. Biol..

[17] Yang J., Zhang L., Yu C., Yang X.F., Wang H. (2014). Monocyte and macrophage differentiation: Circulation inflammatory monocyte as biomarker for inflammatory diseases. Biomark. Res..

[18] Rogacev K.S., Cremers B., Zawada A.M., Seiler S., Binder N., Ege P., Große-Dunker G., Heisel I., Hornof F., Jeken J. (2012). CD14++CD16+ monocytes independently predict cardiovascular events: A cohort study of 951 patients referred for elective coronary angiography. J. Am. Coll. Cardiol..

[19] Afanasieva O.I., Pylaeva E.A., Klesareva E.A., Potakhina A.V., Provatorov S.I., Afanasieva M.I., Krasnikova T.L., Masenko V.P., Arefieva T.I., Pokrovsky S.N. (2016). Lipoprotein(a), its autoantibodies, and circulating T lymphocyte subpopulations as independent risk factors for coronary artery atherosclerosis. Terapevticheskii Arkhiv.

[20] Dahlen G.H., Scanu A.M. (1990). Incidence of Lp(a) among populations. Lipoprotein(a).

[21] Afanas’eva O.I., Adamova I.Y.., Benevolenskaya G.F., Pokrovskii S.N. (1995). Enzyme immunoassay of lipoprotein(a). Bull. Exp. Biol. Med..

[22] Afanas’eva O.I., Klesareva E.A., Levashev P.A., Berestetskaia I.V., Ezhov M.V., Artem’eva N.V., Pokrovskiĭ S.N. (2014). Autoantibodies against lipoprotein(a) in patients with coronary heart disease. Kardiologiia.

[23] Ziegler-Heitbrock L. (2015). Blood Monocytes and Their Subsets: Established Features and Open Questions. Front. Immunol..

[24] Klesareva E.A., Afanas’eva O.I., Donskikh V.V., Adamova I.Y., Pokrovskii S.N. (2016). Characteristics of Lipoprotein(a)-Containing Circulating Immune Complexes as Markers of Coronary Heart Disease. Bull. Exp. Biol. Med..

[25] Sabarinath P.S., Appukuttan P.S. (2015). Immunopathology of desialylation: Human plasma lipoprotein(a) and circulating anti-carbohydrate antibodies form immune complexes that recognize host cells. Mol. Cell. Biochem..

[26] Libby P., Nahrendorf M., Swirski F.K. (2013). Monocyte heterogeneity in cardiovascular disease. Semin. Immunopathol..

[27] Kashiwagi M., Imanishi T., Tsujioka H., Ikejima H., Kuroi A., Ozaki Y., Ishibashi K., Komukai K., Tanimoto T., Ino Y. (2010). Association of monocyte subsets with vulnerability characteristics of coronary plaques as assessed by 64-slice multidetector computed tomography in patients with stable angina pectoris. Atherosclerosis.

[28] Rogacev K.S., Seiler S., Zawada A.M., Reichart B., Herath E., Roth D., Ulrich C., Fliser D., Heine G.H. (2011). CD14++CD16+ monocytes and cardiovascular outcome in patients with chronic kidney disease. Eur. Heart J..

[29] Wrigley B.J., Shantsila E., Tapp L.D., Lip G.Y. (2013). CD14++CD16+ monocytes in patients with acute ischaemic heart failure. Eur J. Clin. Investig..

[30] Krychtiuk K.A., Kastl S.P., Pfaffenberger S., Lenz M., Hofbauer S.L., Wonnerth A., Koller L., Katsaros K.M., Pongratz T., Goliasch G. (2015). Association of small dense LDL serum levels and circulating monocyte subsets in stable coronary artery disease. PLoS ONE.

[31] Krychtiuk K.A., Kastl S.P., Pfaffenberger S., Pongratz T., Hofbauer S.L., Wonnerth A., Katsaros K.M., Goliasch G., Gaspar L., Huber K. (2014). Small high-density lipoprotein is associated with monocyte subsets in stable coronary artery disease. Atherosclerosis.

[32] Krychtiuk K.A., Kastl S.P., Hofbauer S.L., Wonnerth A., Goliasch G., Ozsvar-Kozma M., Katsaros K.M., Maurer G., Huber K., Dostal E. (2015). Monocyte subset distribution in patients with stable atherosclerosis and elevated levels of lipoprotein(a). J. Clin. Lipidol..

[33] Misharin A.V., Cuda C.M., Saber R., Turner J.D., Gierut A.K., Haines G.K., Berdnikovs S., Filer A., Clark A.R., Buckley C.D. (2014). Nonclassical Ly6C(-) monocytes drive the development of inflammatory arthritis in mice. Cell Rep..

[34] Puchner A., Saferding V., Bonelli M., Mikami Y., Hofmann M., Brunner J.S., Caldera M., Goncalves-Alves E., Binder N.B., Fischer A. (2018). Non-classical monocytes as mediators of tissue destruction in arthritis. Ann. Rheum. Dis..

[35] Hirose S., Lin Q., Ohtsuji M., Nishimura H., Verbeek J.S. (2019). Monocyte subsets involved in the development of systemic lupus erythematosus and rheumatoid arthritis. Int. Immunol..

[36] Randolph G.J., Sanchez-Schmitz G., Liebman R.M., Schäkel K. (2002). The CD16(+) (FcgammaRIII(+)) subset of human monocytes preferentially becomes migratory dendritic cells in a model tissue setting. J. Exp. Med..

[37] Cros J., Cagnard N., Woollard K., Patey N., Zhang S.Y., Senechal B., Puel A., Biswas S.K., Moshous D., Picard C. (2010). Human CD14dim monocytes patrol and sense nucleic acids and viruses via TLR7 and TLR8 receptors. Immunity.

[38] Ożańska A., Szymczak D., Rybka J. (2020). Pattern of human monocyte subpopulations in health and disease. Scand. J. Immunol..

[39] Urbanski K., Ludew D., Filip G., Filip M., Sagan A., Szczepaniak P., Grudzien G., Sadowski J., Jasiewicz-Honkisz B., Sliwa T. (2017). CD14+CD16++ “nonclassical” monocytes are associated with endothelial dysfunction in patients with coronary artery disease. Thromb. Haemost..

[40] Kral B.G., Kalyani R.R., Yanek L.R., Vaidya D., Fishman E.K., Becker D.M., Becker L.C. (2016). Relation of Plasma Lipoprotein(a) to Subclinical Coronary Plaque Volumes, Triple-vessel and Left Main Coronary Disease, and Severe Coronary Stenoses in Apparently Healthy African-Americans With a Family History of Early-Onset Coronary Artery Disease. Am. J. Cardiol..

[41] Pirro M., Bianconi V., Paciullo F., Mannarino M.R., Bagaglia F., Sahebkar A. (2017). Lipoprotein(a) and inflammation: A dangerous duet leading to endothelial loss of integrity. Pharmacol. Res..

[42] Wong K.L., Tai J.J., Wong W.C., Han H., Sem X., Yeap W.H., Kourilsky P., Wong S.C. (2011). Gene expression profiling reveals the defining features of the classical.; intermediate, and nonclassical human monocyte subsets. Blood.

[43] Zawada A.M., Rogacev K.S., Rotter B., Winter P., Marell R.R., Fliser D., Heine G.H. (2011). SuperSAGE evidence for CD14++CD16+ monocytes as a third monocyte subset. Blood.

[44] Afanasieva O.I., Utkina E.A., Artemieva N.V., Ezhov M.V., Adamova I.Y., Pokrovsky S.N. (2016). [Elevated Lipoprotein(a) Cncentration and Presence of Subfractions of Small Dense Low Density Lipoproteins as Independent Factors of Risk of Ischemic Heart Disease]. Kardiologiia.

[45] Yamamoto H., Yoshida N., Shinke T., Otake H., Kuroda M., Sakaguchi K., Hirota Y., Toba T., Takahashi H., Terashita D. (2018). Impact of CD14++CD16+ monocytes on coronary plaque vulnerability assessed by optical coherence tomography in coronary artery disease patients. Atherosclerosis.

