# Supplementary material for: The Association of Lipoprotein(a) and Circulating Monocyte Subsets with Severe Coronary Atherosclerosis
Source: J Cardiovasc Dev Dis. 2021 Jun 1;8(6):63. doi: 10.3390/jcdd8060063 (PMC8228191; doi:10.3390/jcdd8060063)
Supplement: Supplementary file 1 [file jcdd-08-00063-s001.zip › jcdd-1210444-supplementary.pdf]

Supplement materials

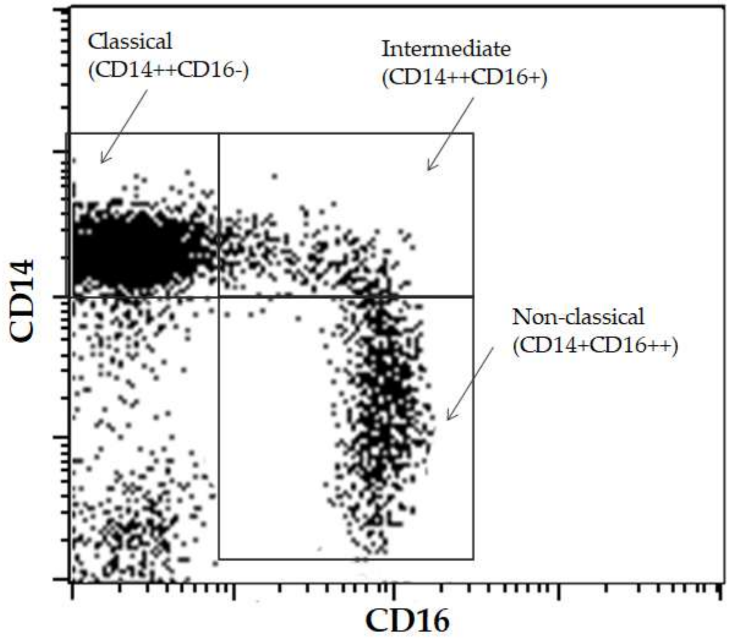

Figure S1. Monocyte subsets definition based on a typical distribution of events in a CD14 and CD16 staining. Flow cytometry dot plot showing the gating of the classical CD14++CD16-, intermediate CD14++CD16+ and nonclassical CD14+CD16++ monocyte subsets. Classical monocytes express high levels of CD14 but no CD16, intermediate monocytes express high levels of CD14 and low CD16, while non-classical monocytes express low CD14 but high CD16.

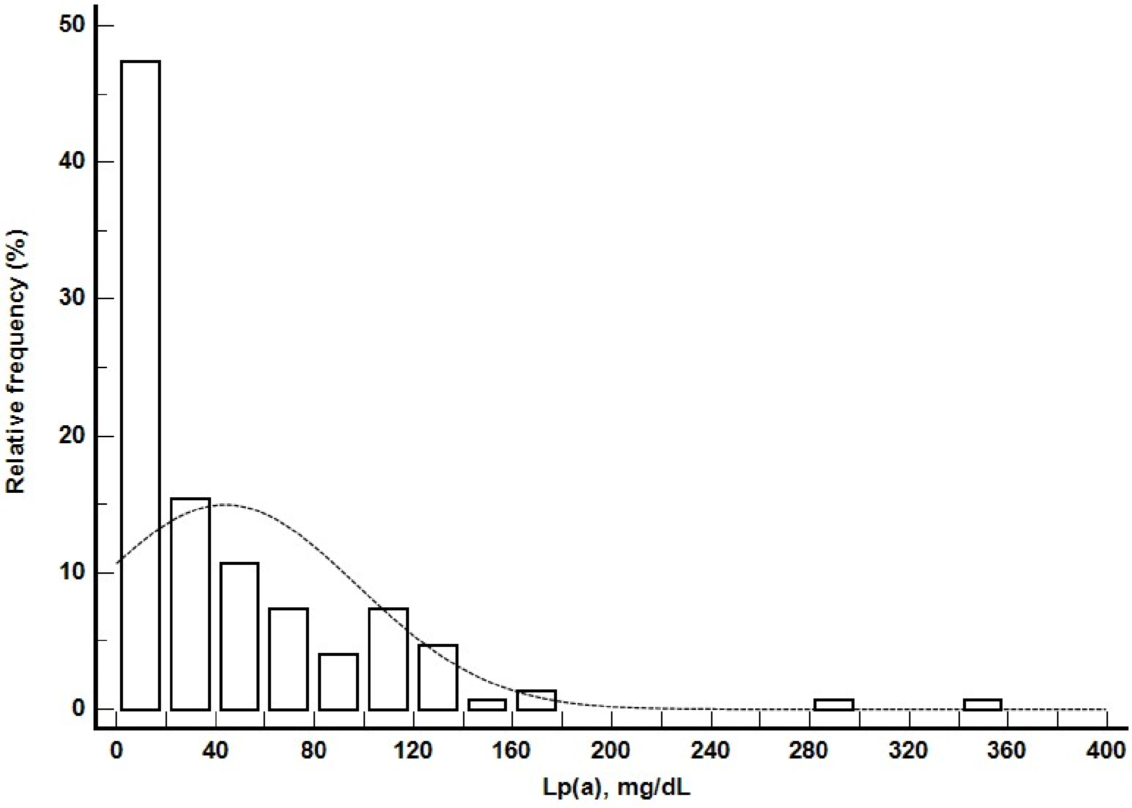

Figure S2. Histogram of the distribution of the Lp(a) concentration in the examined patients.
This information was added in limitation of the study and Fig S2 was added in Supplement materials.

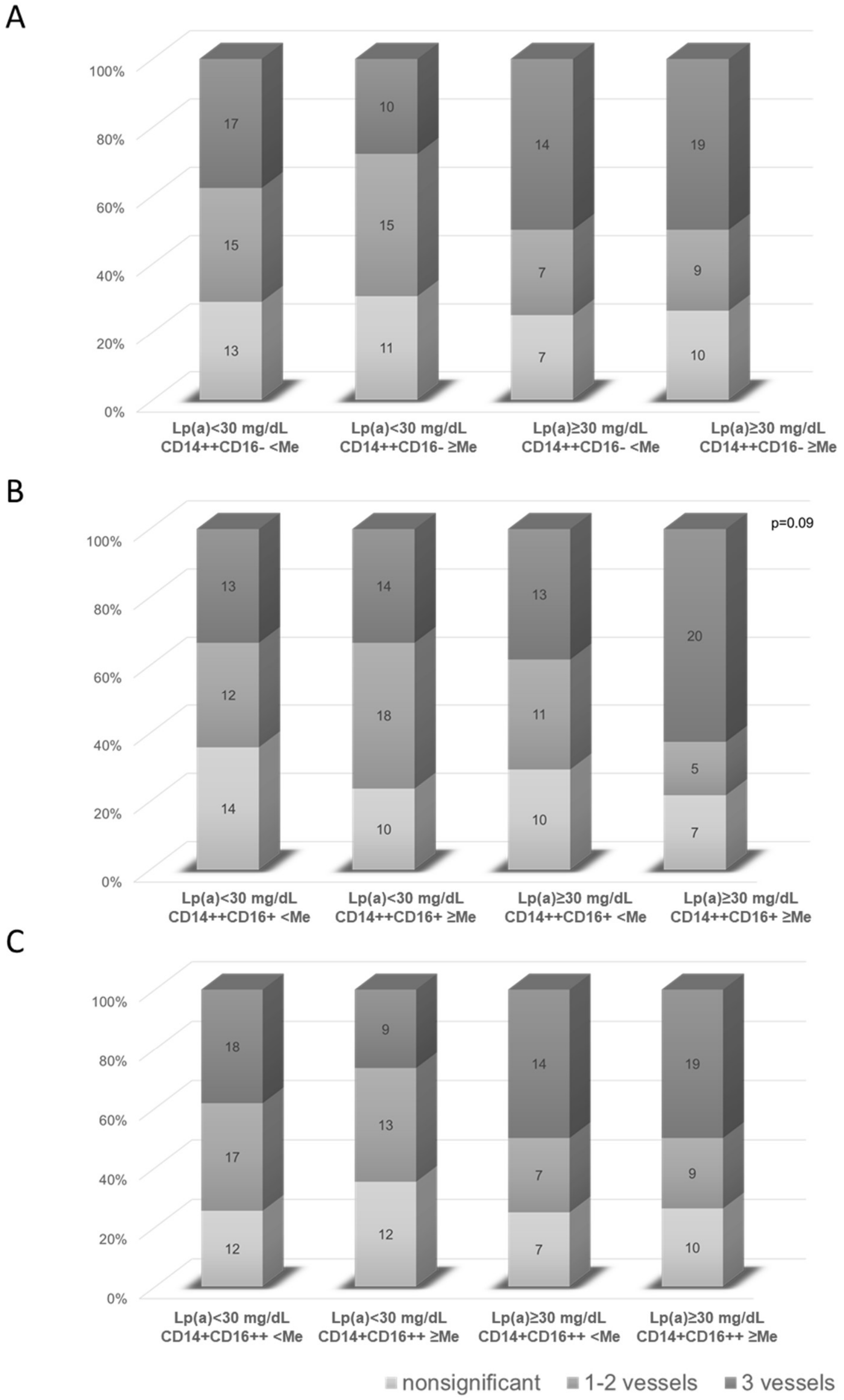

Figure S3. Severity of coronary artery disease depending on the presence of hyperLp(a) and absolute content of classical CD14++CD16- (A), intermediate CD14++CD16+ (B) and non-classical CD14+CD16++ monocytes (C). The data are presented as the percentage of patients and the absolute number of patients (numbers within the bars) with different severity of coronary atherosclerosis in subgroups, depending on the combination of normal (<30 mg/dL) and increased (≥30 mg/dL) concentrations of Lp(a), as well as the absolute content of monocytes below and above the median. The corresponding values of the median for absolute content of classical CD14++CD16-, intermediate CD14++CD16+ and nonclassical CD14+CD16++ is subpopulations of monocytes were 327.4×10^3/mL, 32.5×10^3/mL and 70.2×10^3/mL, respectively.
